# *Saccharina latissima* Cultivated in Northern Norway: Reduction of Potentially Toxic Elements during Processing in Relation to Cultivation Depth

**DOI:** 10.3390/foods10061290

**Published:** 2021-06-04

**Authors:** Marthe Jordbrekk Blikra, Xinxin Wang, Philip James, Dagbjørn Skipnes

**Affiliations:** 1 Department of Processing Technology, Seafood Division, Nofima AS, P.O. Box 8034, NO-4068 Stavanger, Norway; dagbjorn.skipnes@nofima.no; 2 Department of Aquaculture Production, Aquaculture Division, Nofima AS, P.O. Box 6122, NO-9291 Tromsø, Norway; xinxin.wang@nofima.no (X.W.); philip.james@nofima.no (P.J.)

**Keywords:** *Saccharina latissima*, seaweed, processing, cooking, cultivation depth, iodine, heavy metals, potentially toxic elements, northern Norway, tolerable dietary intake

## Abstract

There is an increasing interest in the use of *Saccharina latissima* (sugar kelp) as food, but the high iodine content in raw sugar kelp limits the daily recommended intake to relatively low levels. Processing strategies for iodine reduction are therefore needed. Boiling may reduce the iodine content effectively, but not predictably, since reductions from 38–94% have been reported. Thus, more information on which factors affect the reduction of iodine are needed. In this paper, sugar kelp cultivated at different depths were rinsed and boiled, to assess the effect of cultivation depth on the removal efficacy of potentially toxic elements (PTEs), especially iodine, cadmium, and arsenic, during processing. Raw kelp cultivated at 9 m contained significantly more iodine than kelp cultivated at 1 m, but the difference disappeared after processing. Furthermore, the content of cadmium and arsenic was not significantly affected by cultivation depth. The average reduction during rinsing and boiling was 85% for iodine and 43% for arsenic, but no significant amount of cadmium, lead, or mercury was removed. Cultivation depths determined the relative effect of processing on the iodine content, with a higher reduction for kelp cultivated at 9 m (87%) compared to 1 m (82%). When not taken into consideration, cultivation depth could mask small reductions in iodine content during rinsing or washing. Furthermore, since the final content of PTEs was not dependent on the cultivation depth, the type and extent of processing determines whether cultivation depth should be considered as a factor in cultivation infrastructure design and implementation, or alternatively, in product segmentation.

## 1. Introduction

Seaweeds are attracting increasing interest as a food source in European and other Western countries. It is well documented that global climate gas emission levels can be reduced by cultivation of seaweeds [1], and thus seaweed consumption may be considered an ethical choice to many consumers [2,3]. Seaweeds can also provide new and interesting tastes and eating experiences for western consumers, where seaweed is considered a new or rediscovered food or food component [4].

In Northern Europe, the brown alga *Saccharina latissima* (sugar kelp) is one of the most popular species for cultivation and it is slowly making an appearance in the food market, starting with health food stores, fine food stores, fish markets, and high-end Asian restaurants. Despite several other species being better choices with respect to nutrition [5], *S. latissima* has become popular due to its relatively stable and high production compared to other macroalgae [6]. An abundance of minerals and bioactive, health promoting components are found in *S. latissima*, and the structure is built from indigestible dietary fibers [7,8]. The main bulk of the fiber is alginate, which is soluble and can increase satiety after consumption and thereby limit subsequent food intake [9].

Two bottlenecks are currently limiting the intake of seaweed in the European population. The first is ‘food neophobia’, which means skepticism in large parts of the population to including new raw materials in their diet [2]. The second is the content of potentially toxic elements (PTEs) in the seaweed, especially iodine, arsenic, cadmium, lead, and mercury. This latter point will be addressed in the current study. The high content of PTEs can be tackled either from a breeding and cultivation point of view or from a process technology point of view. In this paper, we will focus on a combination of one of the cultivation conditions (the depth) and the effect of this after standard processing.

Kelps contain an abundance of iodine, with values ranging from 380 to 7200 mg/kg of dry weight in sugar kelp [10,11,12,13,14,15,16]. Iodine is an essential nutrient in the human diet, needed for production of thyroid hormones and for optimal growth, especially in neonates and children. Iodine deficiency is the World’s number one cause of preventable brain damage [17]. The general dietary requirement of adults is 150 μg iodine/day, with higher values for pregnant and lactating women and lower, age-dependent values for children [18]. The maximum daily recommended limit for adults is 600 μg iodine/day in Europe [19]. The risk when adding sugar kelp or other brown algae to the diet is therefore not iodine deficiency but excessive iodine consumption [20]. Following excessive consumption of iodine, some individuals develop thyroid dysfunction disorders, such as hypo- and hyperthyroidism [21]. Excessive, long term consumption of iodine increases the risk of thyroid dysfunction disorders [22]. Both deficient and excessive iodine consumption has serious consequences for fetal growth [23]. Maintaining a balanced iodine status is therefore important in a broader public health perspective. 

The iodine status in European populations is on average iodine insufficient, with some population groups suffering from mild to moderate iodine deficiency, while others are iodine sufficient [17,24]. Vegans are especially prone to iodine deficiency since—apart from seaweeds—there are no good iodine sources in a vegan diet [24,25]. On the other hand, a diet rich in white fish and dairy provides more than sufficient iodine. As an example, according to the Norwegian National Food Composition Database [26], one portion of cod (100 g), 2 dL of milk, and 2 dL of yoghurt provide in total 340 μg of iodine. Since the iodine status ranges from insufficient to adequate, the content of iodine in foods containing seaweed must not be exaggerated, since this provides a real risk of overconsumption of iodine.

The content of PTEs in brown algae is dependent on species and may also vary greatly within one species, depending on factors such as growth conditions and location [10,11,13,27]. Sugar kelp may contain substantial contents of cadmium (0.13–4.6 mg/kg dw) and relatively high doses of total arsenic (26–120 mg/kg dw), while the contents of lead and mercury are usually less problematic [12,15,27,28,29,30,31,32,33,34]. As pointed out in the recent report by Duinker and Kleppe et al. [10], more knowledge of the factors causing variations in the content of PTEs of seaweeds are needed to allow predictable product quality.

Like iodine, any additional arsenic, cadmium, lead, and mercury added to the diet with new foods will add to the existing PTE burden already present in the diet. Thus, not only should the PTE content of the new food (seaweed) be taken into account, but also that of food in the preexisting diet of a given population. Cadmium, lead, mercury, and inorganic arsenic pose a risk of toxicity and can increase the risk of cancer [35,36,37,38]. Therefore, they have acute dietary limitations. This holistic view of seaweeds as part of the diet instead of as a single food item is beyond the scope of this paper, but this paper will provide data for such future considerations.

Since some components, especially iodine and arsenic, can be reduced by preparation and cooking methods, the safety of consuming seaweeds can be increased by processing prior to consumption. Iodine reduction up to 94% has been reported during blanching of sugar kelp [16], and a reduction of 38% was found during boiling in another study [15]. Thus, although the iodine content of sugar kelp may be reduced, the degree of reduction cannot be said to be predictable until more knowledge of the factors causing these differences are in place. The same applies to the reduction of arsenic during cooking. Some studies have reported a reduction in the arsenic content during cooking of brown algae [39,40,41], while in other cases no reduction occurred [15,39]. Only one of these studies investigated cooking of *S. latissima*. More species-specific data is also required since different species may respond differently to the processing conditions applied.

Any processing performed on foods should aim to reduce the content of undesired components in the food while maintaining or improving desirable traits such as nutritive value. Previous studies have shown that blanching and boiling does not reduce the protein or lipid content of *S. latissima*, although the ash content decreases [15,16]. In the case of blanching, the phenolic content and radical scavenging activity increased after processing compared to raw kelp [16]. However, the content of two non-essential amino acids, glutamic acid and alanine, was reduced during blanching. Although this did not affect the nutritional profile of the kelp, this may affect the taste of the kelp, since glutamic acid is associated with the fifth taste, “umami”, which signifies a “unique and savory taste sensation” [4]. Thus, in addition to improving the safety of seaweed consumption while retaining nutritional value, boiling and blanching may reduce the intensity of the seaweed taste. It is up to the consumers and the further application of kelp to determine whether this is a desirable or non-desirable change.

The aim of this study is to contribute to resolving issues related to the undesired high levels of PTEs in sugar kelp for food. To the best of our knowledge, this is the first study investigating the effect of cultivation depth on the reduction in iodine, arsenic, cadmium, lead, and mercury content of sugar kelp after processing. Furthermore, this study provides data on the content of PTEs in *S. latissima* from a more northern location in Norway than what has been previously reported in the scientific literature. 

## 2. Materials and Methods

### 2.1. Sporeling Production

**Figure 1:**
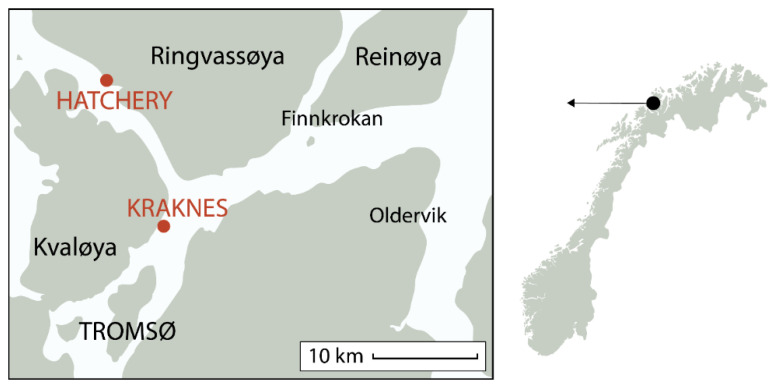
Map of hatchery and cultivation site (Kraknes).

*Saccharina latissima* sporophytes were collected from Kraknes (Figure 1) in January 2020 and brought back to the seaweed laboratory in Nofima, Tromsø. The Kraknes site is a research area, run by Akvaplan-Niva. The site is located on the island of Kvaløya (69° 45.259′ N/019° 02.176′ E) near Tromsø, with a water depth of 15–20 m. This site has well-mixed water masses through tidal forcing. The main current direction was towards the northwest. According to measurements at 12 m depth from March to July 2011, the current velocity is moderate, with an average of 3.4 cm/s and a maximum of 22 cm/s [42]. No significant changes have been made to the area (e.g., building of a breakwater, pier or similar) since these measurements were taken.

Ripe sori tissue from 15 sporophytes was punched out and used for the production of sporeling lines. Sporelings were produced in the seaweed hatchery in Nofima, Tromsø, according to Forbord and Steinhovden et al. [43] with minor revisions. A solution of approximately 250,000 spores/mL seawater was brushed onto 1.2 mm diameter twine coiled around PVC spools. The spools were then incubated for 10 weeks in seawater from 50 m depth in a flowthrough (120 L/h) cylinder (150 cm high and 50 cm diameter) at 10 °C. LED lights were placed both in and outside the cylinders, with a light intensity of 20 μmol photons m^2^/s initially with a gradual increase to 70 μmol photons m^2^/s by the end of the incubation period. The seawater temperature was kept at about 10 °C during the first 6 weeks and then gradually decreased to match the ambient seawater temperature.

### 2.2. Deployment and Growout at Sea

When the sporelings reached an average length of approximately 0.5 cm, the twines were entwined onto ropes (10 m long and 10 mm thick) and packed in polystyrene boxes. In March 2020, five ropes were vertically deployed approximately 2 m from one another at Kraknes where the sori were collected.

### 2.3. Sampling

*Saccharina latissima* sporophytes from 0–2, 5–7, and 8–10 m depth intervals were harvested in June 2020, packed in polystyrene boxes, and transported to the seaweed laboratory. For simplicity, these depths are referred to throughout the paper as 1, 6, and 9 m. The average frond lengths of *S. latissima* cultivated at 1, 6, and 9 m, based on a sub-sample of 20 specimens per depth, were 121 ± 28, 132 ± 23, and 111 ± 29 cm, respectively. The average wet weights of the same samples were 84 ± 47, 101 ± 59, and 75 ± 43 g, respectively. There were no significant differences in weight and length between the sample groups. The average frond length and wet weight for all the samples collectively was 120 ± 28 cm and 85 ± 49 g, respectively. Samples were stored at −30 °C until further analysis.

For analysis of PTEs in raw kelp, 30 *S. latissima* sporophytes from each depth interval were thawed at room temperature and the whole thallus was dried at 25 °C in an oven for 72 h. Samples were ground in a coffee mill for about 5 min and pooled together. Subsamples of about 10 g were taken for iodine analyses.

### 2.4. Cooking Experiment

The kelp was thawed at 0–2 °C overnight. The following day, the holdfasts and stipes were cut away from the blade and approx. 700 g of blades were put in a 29.5 × 48.8 cm tray and cold water was added (approx. 5 cm, amounting to approx. 7 L). The seaweed was gently moved around by gloved hands and the water was changed twice (=3 times rinsing). Then, the blades were placed on a perforated tray to allow water to drip from the blades for 10–15 min. Following this, 511 ± 5 g of sugar kelp was boiled in 5 L water for 15 min. The initial temperature of the water was 99.3 ± 0.4 °C and after the seaweeds were placed in the cooking pot, the temperature dropped to 92.2 ± 3.3 °C. The temperature was restored to 99 °C again after 7.4 ± 2.0 min. After cooking, the seaweeds were sieved and immediately placed in a 10 L bucket of water and ice and stirred to enable cooling uniformity for 0.5–1 min prior to sieving again. The sugar kelp was then placed in 1–3 layers on a baking plate. Prior to this, the baking plate was covered with baking paper to keep the seaweed from sticking to the plate after drying. Drying was performed in a smoking chamber at 25 °C for 72 h. After the drying was complete (4 days after thawing was initiated), a second parallel cooking trial was performed where the order of samples was reversed. The dry samples were subsequently ground in a mini-food processor (until approx. 1 mm in size) before sub-sampling for analyses.

### 2.5. Iodine Analysis

Elemental iodine analysis was performed by Mikroanalytisches Labor Kolbe, Oberhausen, Germany. Ground seaweed samples (*n* = 3) were crushed using an IKA MF10 mill and taken through a 0.5 mm sieve. The digestion was done in a special combustion unit from A1-Envirosciences (AQF-2100) with a manual sampler, at 1100 °C and burned in an argon/oxygen stream. The resulting gases were measured on a Metrohm Model 883 Plus ion chromatograph (IC). The lower limit of detection of iodine in the dried seaweed samples was determined according to the calibration line method, as the smallest amount of a sample containing an α-error of 5% and a β-error of 50% that can be proven qualitatively. The detection limit was determined based on the sample mass and the detection limit for the determination of iodine in the IC and was 1 ppm. For control purposes, a control sample (an organic compound containing a defined amount of iodine) was burned and measured at regular intervals to make sure that there were no errors. The analytical standard deviation of the measurements was 0.15% of the measured values.

### 2.6. Analysis of Arsenic, Cadmium, Lead and Mercury

Analysis of arsenic, cadmium, lead, and mercury was performed by ALS Scandinavia AB Luleå Aurorum 10, Sweden, following SS-EN 13805:2014 and using accredited methodology, as described below.

#### 2.6.1. Sample Preparations

To allow uniform blind handling, all samples were given a code laboratory number. Sample preparation was performed in Class 10,000 clean laboratory areas by personnel wearing clean room attire. General precautions detailed by Rodushkin and Engström et al. [44] were taken to minimize contamination. Laboratory materials used during sample preparation were soaked in 0.7 M nitric acid for 24 h at room temperature and rinsed with de-ionized Milli-Q water prior to use. Samples (approx. 1 g) were weighed into 50 mL polypropylene tubes, 10 mL of nitric acid, and 0.05 mL of hydrofluoric acid (Suprapur grade) were added, the tubes were loosely cupped and left inside a fume hood at room temperature overnight. The following day, sample digestion was performed in a graphite heating block at 110 °C for 120 min. Digests were diluted to 30 mL with Milli-Q water, homogenized by agitation, and further diluted with 1.4 M nitric acid to provide a total dilution factor of 1000 (v/m). Two preparation blanks, two duplicate samples and two reference materials were included in each preparation batch of 36 samples.

#### 2.6.2. Instrumental Analysis

Concentrations of all elements were determined by double-focusing, sector field inductively coupled plasma mass-spectrometry (ICP-SFMS, ELEMENT XR, Thermo Scientific, Bremen, Germany) operated with methane addition to the plasma [45] and equipped with a solution nebulization sample introduction system. Matrix effect correction was accomplished by internal standardization by adding indium to all measurement solutions at 2.5 µL/L concentration. Quantification was done by external standardization with synthetic, concentration-matched standards. Further details on the operation conditions and measured parameters as well as figures of merit of the method are described by Engström and Stenberg et al. [46]. The limits of detection (LOD) were calculated as three times the standard deviation for element concentrations detected in the preparation blanks (*n* > 15).

#### 2.6.3. Quality Control and Quality Assurance Procedures

The accuracy of the ICP-SFMS data was assessed by analyses of the two certified reference materials (CRMs), namely tea (GBW 07605; Institute of Geophysical and Geochemical Exploration. Langfang, China) and wheat flour (NBS 1467a; NIST, Gaithersburg, MD, USA). These CRMs were selected on the basis of expected similarities in matrix and elemental concentrations. The ICP-SFMS results were within 10% of the relative standard deviation (RSD) range of certified, indicative, or information values, when such were available. Method reproducibility was evaluated from results for duplicate samples. To account for as many sources of deviation as possible. Samples containing the highest concentrations of arsenic, cadmium, lead, and mercury underwent repeat testing when first tested.

### 2.7. Dry Matter Analysis

Dry matter analysis was performed on dried samples to assess the residual moisture. Duplicate samples of 0.9 ± 0.3 g were weighed into pre-weighted aluminum cups and subsequently dried at 105 °C for 18–20 h. The content of PTEs is expressed per g dry sample with the residual moisture content not accounted for instead of per g dry matter. In this way, the tolerable amounts of dried kelp can be expressed per g dry sample and thus utilized directly when weighing the amount of kelp which can safely be consumed.

### 2.8. Tolerable Daily Amounts of Saccharina latissima

Based on the experimental data, the amount of *S. latissima* providing 150 and 600 μg of iodine was calculated. The amounts were used as the baseline for calculations of arsenic, cadmium, lead, and mercury in boiled, dry sugar kelp. Tolerable weekly intakes (TWI) and tolerable daily intakes (TDI) of kelp were calculated per kg body weight (bw) and for a 70 kg person, based on the limits proposed by EFSA [35,36,37,38].

### 2.9. Statistics

Analysis of variance (ANOVA) was performed to test for significant differences between sample groups, using Minitab^®^ version 19.2020.1 and a 95% confidence interval. A Tukey post hoc test was applied when more than two sample groups were present. The results are given as average ± standard deviation, where the standard deviation (σ) was calculated according to Equation (1), i.e., the sample standard deviation.

(1) $$\sigma =\sum \frac{{\left(x-\bar{x}\right)}^{2}}{n-1}$$

## 3. Results

### 3.1. Iodine

#### 3.1.1. Effect of Cultivation Depth

The analyzed samples had an average iodine content of 4100 ± 700 μg/g dw. Cultivation depth had a significant effect on the iodine content of raw kelp and higher levels of iodine were recorded for kelp grown at deeper depths than the kelp grown closer to the sea surface (Table 1). After rinsing, the kelp cultivated at 9 m still had significantly more iodine than the kelp cultivated at 1 and 6 m, but after boiling no significant differences were found. Thus, the effect of cultivation depth on the iodine content was pronounced for raw samples but disappeared after boiling.

#### 3.1.2. Effect of Processing

When considering data for all depths collectively, boiling significantly reduced iodine content compared to raw and rinsed kelp by 85%. However, rinsing alone did not reduce iodine content compared to raw kelp.

When analyzing each cultivation depth separately, rinsing also significantly reduced the iodine content of kelp cultivated at 6 and 9 m, but not for kelp cultivated at 1 m.

### 3.2. Arsenic, Cadmium, Lead and Mercury

Processing decreased arsenic content significantly but not the content of cadmium and lead (Table 1). The content of arsenic in raw kelp was 62.7 ± 4.3 mg/kg dw and it was reduced by rinsing and boiling to 36.0 ± 3.1 mg/kg dw. For mercury, the content increased slightly but significantly after rinsing compared to raw kelp, but after boiling the content was not significantly different compared to the content in raw kelp, reaching a final concentration of 0.0280 ± 0.0032 mg/kg dw. The cadmium and lead contents were not dependent on processing. The weighted mean concentration of cadmium and lead were 2.00 ± 0.41 and 0.852 ± 0.878 mg/kg dw, respectively.

Cultivation depth showed no significant effect on the content of arsenic, cadmium, lead, or mercury, although some trends appeared. A consistent increase in cadmium content with deeper cultivation depth was observed both for raw and processed kelp. The same tendency was observed for arsenic content although not for boiled kelp. Due to a lack of statistical significance, these results should be interpreted with caution unless validated in a separate study.

### 3.3. Tolerable Daily Amounts of Saccharina latissima

The amount of *S. latissima* providing the daily requirement (150 μg) and the maximum daily recommended intake (600 μg) of iodine for adults was 0.25 and 1 g of boiled, dried kelp (Table 2). The content of arsenic, cadmium, lead, and mercury in boiled, dried sugar kelp in these amounts make up 0.1–8.7% of the TDI of these PTEs for a 70 kg person, with cadmium being the PTE associated with the highest percentage (Table 3).

The TWI of cadmium was set to 2.5 μg/kg body weight (bw) [38], which gives a TDI of 25 μg for a 70 kg person. Eating 1 g of boiled, dried sugar kelp will provide 2.19 μg of cadmium, equaling 8.7% of the TDI.

For lead, several benchmark dose lower confidence limits (BMDL) were determined by EFSA, the lowest of which represents a 1% extra risk for developmental neurotoxicity in children under the age of seven, at BMDL_01_ of 0.5 μg/kg bw [37]. Despite tolerable amounts being calculated for adult persons in this work, this limit was still chosen as a precautionary measure. This limit gives a TDI of 35 µg for a 70 kg person. Thus, eating 1 g of boiled, dried sugar kelp exposes the person to 3.3% of the TDI.

There are no tolerable limits set for total arsenic in food. For inorganic arsenic, the BMDL representing 1% extra risk for disease, BMDL_01_, was set at 0.3–8 μg/kg bw/day [35]. Applying the precautionary principle, the limit of 0.3 μg/kg bw/day is used herein. The inorganic fraction of arsenic in sugar kelp was not measured in this study but has previously been found to range between 0.4–0.9% of total arsenic [12,28]. Reduction of inorganic arsenic from sugar kelp during processing has, to the best of the knowledge of the authors, not been characterized and so we assume that the content of inorganic arsenic does not change during processing. The arsenic content in raw kelp, 63 mg/kg dw, was therefore used to estimate the amount of inorganic arsenic. Assuming a 1% fraction of inorganic content, this yields 0.63 µg of inorganic arsenic per 1 g of dry seaweed sample. Based on the limit of 0.3 μg/kg body weight per day, the TDI of inorganic arsenic from foods is 21 µg for a 70 kg person. Thus, 1 g of dried sugar kelp contain a maximum of 3% of the TDI of inorganic arsenic.

For mercury, a TWI of 4 μg/kg bw is given for inorganic mercury (iHg) and 1.3 μg/kg bw for methyl mercury (MeHg) [36]. Assuming that all the mercury in the sample was iHg, 1 g of boiled, dried *S. latissima* exposes the consumer to <0.1% of the TDI. If assuming instead that all the mercury in the sample was MeHg, 1 g of boiled, dried *S. latissima* exposes the consumer to 0.2% of the TDI. Methyl mercury has, to the best of our knowledge, only been characterized in *S. latissima* in one study, where it was found to be below the limit of detection [28].

## 4. Discussion

### 4.1. Potentially Toxic Elements

#### 4.1.1. Iodine

The iodine content in raw *S. latissima* was on average 4100 ± 700 mg/kg dw in the present study, and the results ranged from 3300–4800 mg/kg dw. These results are consistent with previously reported values for the same species, which vary from 380–7200 mg/kg dw [10,11,12,13,14,15,16,34].

In our study, the cultivation depth had a significant impact on the iodine content in raw kelp, with higher iodine content in kelp cultivated at deeper depths. The iodine content in raw kelp cultivated at 1 m-depth was 3300 ± 0 mg/kg dw, compared to 4700 ± 100 mg/kg dw for cultivation at 9 m-depth. A previous study analyzing *S. latissima* cultivated close to Frøya (an Island near Trondheim), also investigated the effect of cultivation depth (3 and 8 m) on the iodine content of *S. latissima* [34]. In the aforementioned study, samples were harvested in May, June, and August. In May, no difference was seen in the iodine content of samples cultivated at 3 compared to 8 m, whereas in June, samples cultivated at 8 m had a slightly higher content (4200 mg/kg dw) compared to those cultivated at 3 m (3900 mg/kg dw). In August, an opposite trend appeared. No statistical significance could be drawn from the data since all analyses were performed in a single parallel. However, based on the data presented here, it seems that cultivation depth has a greater impact on the iodine content of raw sugar kelp cultivated in the north of Norway (near Tromsø) compared to in the middle of Norway (near Trondheim). This could be either due to genetic heterogeneity [47], or a result of differences in environmental conditions between the locations [13]. 

The difference in iodine content between cultivation depths was no longer apparent after rinsing and boiling. The additional iodine in the raw kelp cultivated at 6 and 9 m compared to 1 m therefore seemed to be easily extractable from the matrix using the applied processing conditions and can be assumed to be water soluble. Iodine can be found in many chemical forms in seaweed, but in species in the Laminariaceae family (including *Saccharina* spp. and *Laminaria* spp.), most is iodide (I^−^). The content of iodide in *Saccharina japonica* was found to be 88–94% [48,49], while 99.2% of the iodine in *S. japonica* was water soluble [48]. For *S. latissima*, an iodide content of 574 mg/kg dw has been found [50]. In the latter study, the total iodine content was not measured, so the proportion of iodide as a fraction of total iodine cannot be estimated. To the best of our knowledge, no other studies have investigated the iodide content of *S. latissima*. Based on the results from the present study, it seems that the water extractable fraction of iodine from sugar kelp cultivated at greater depths was higher compared to the sugar kelp cultivated at shallower depths, whereas the content that was not easily extracted was similar. This explains why there were no significant differences between cultivation depths after processing. The importance of taking cultivation depth into account therefore seems to depend on the processing conditions the kelp is exposed to prior to human consumption. 

In the present study, the iodine content was reduced on average by 85% from 4100 to 600 mg/kg dw by rinsing three times and boiling for 15 min, when taking all cultivation depths into account. The relative reduction was greater for kelp cultivated at 9 m (87%) compared to at 1 m depth (82%). Other studies have also assessed the effect of processing on the iodine content of cultivated sugar kelp, but they did not describe the cultivation depths at which the kelp was grown. The effect of boiling and blanching treatments on iodine reduction from sugar kelp varies from 38–94%, leaving final iodine contents from 87.4 to 1620 mg/kg dw [11,15,16]. Cultivation depth may play a role in the effect of processing and when not taken into consideration, cultivation depth could mask small reductions in iodine content during processing steps such as rinsing or washing.

In addition to cultivation depth, another factor that could influence the relative iodine reduction is the water to seaweed ratio during boiling/blanching. In non-living kelp, iodine reduction during soaking, blanching, and boiling is likely a diffusion driven process, where the content of iodine is transported from a system of high concentration (the kelp) to a system of low concentration (the water). During repeated soaking of *S. japonica* in distilled water at room temperature, 84.6% of the iodine was removed in the first soaking (3 h), an additional 13.6% during the second (30 min), after the water was replaced, and 1% in the third (30 min) [48]. For *S. latissima*, a high water-to-kelp ratio (33 times more water than seaweed) was applied in the blanching process (120 s at 80 °C), which yielded a 94% iodine reduction [16]. A low ratio, only four times more water than kelp, resulted in a 38% iodine reduction during boiling for 15 min [15]. In both cases, local tap water was used. Intermediate ratios yielded results between these extremes (Table 4). 

The initial water quality could also be equally relevant for iodine reduction. The lowest reduction during treatment of *S. latissima* in water was found in a study performed in Denmark using tap water [15]. Since the tap water in Denmark has been known to contain significant amounts of humid iodine substances [51], the iodine content in the water prior to the experiment could be a co-determining factor for the low iodine reduction found in the study by Bruhn and Brynning et al. [15] compared to the other studies discussed above. In future studies it could therefore be worth checking the effect of using distilled water compared to the effect of tap water, since local differences in the tap water quality may be a co-determining factor in the iodine reduction.

A final factor that could influence the iodine reduction is the part of the thallus used. In our study, the holdfasts and stipes were removed prior to rinsing and boiling but kept for samples prepared for analysis of PTEs in raw kelp. This could be of importance for the data interpretation, since the iodine content of *S. latissima* was previously shown to vary with thallus part. Nitschke and Stengel [52] showed that the holdfast of *S. latissima* contained similar contents of iodine to the stipe, which was higher than the content of the blade and meristem (per dw). Kreissig and Hansen et al. [53] confirmed a higher content of iodine in stipes than the blade of this species. Thus, when we removed the stipes and holdfast prior to processing, we removed a part of the kelp that is higher in iodine content than the remaining part. Even though these parts are small compared to the blade, this could influence the result.

#### 4.1.2. Arsenic

In this study, the total arsenic content of raw sugar kelp was 62.7 ± 4.3 mg/kg dw, which is in line with previous studies reporting arsenic concentrations between 26–120 mg/kg dw in *S. latissima* [12,15,27,28,29,30,31,32,33,34].

Arsenic occurs as many chemical forms and a distinction between organic and inorganic arsenic is often made. Inorganic arsenic is considered the most toxic form of arsenic and it is carcinogenic. The toxicity of organic arsenic forms in seaweeds, most notably arsenosugars, are not fully characterized. Based on similarities with other arsenosugars and organic arsenic species, they are encouraged to be considered “potentially toxic” [54,55]. The fraction of inorganic arsenic in sugar kelp was not analyzed in this study, but is generally very low, with values ranging from 0.16–0.39 mg/kg dw [12,14,28], which makes up <1% of the total arsenic content.

The results of arsenic removal during the processing of sugar kelp are contradictory. In this study, the arsenic content was significantly reduced by rinsing three times in water (−18.9%), and the content was further reduced, although not significantly, by boiling for 15 min, achieving a total reduction of 42.5% and a final concentration of 36.0 ± 3.1 mg/kg dw. In another study, boiling frozen-thawed *S. latissima* from the Faroe Islands increased the arsenic content significantly from 39 to 42 mg/kg dry weight [15]. In some cases, boiling in water containing arsenic can increase the content of arsenic in foods [55], but a loss of other dry matter components, such as iodine, may also yield this result. Subsequent fermentation did not alter the arsenic content compared to unprocessed sugar kelp [15].

For other brown algae, both total arsenic and inorganic arsenic have been found to decrease during processing in water, although like for *S. latissima*, one study found no reduction (Table 5). The boiling of dry, commercial samples of *Undaria pinnatifida* for 20 min did not reduce the arsenic content per dry matter in one study [39], whereas in another study the arsenic content was reduced (−49%) following the same treatment [56]. Boiling 15 g of a dry, commercial sample of *S. japonica* in 300 mL of milli-Q water for 60 min reduced the total arsenic content by 65%, yielding an arsenic content of 27.2 mg/kg dw after processing [56]. Similarly, the boiling of dry, commercial samples of *Hizikia fusiforme* reduced the arsenic content by 30–46% [39,40]. Blanching of dry samples of *L. digitata* and *S. japonica* for 10–20 s reduced the arsenic content by 22–39% [41]. In samples washed before drying, the majority of reduction in arsenic was due to reduction in organic but not inorganic arsenic, while in samples not washed before drying both organic and inorganic species were lost in roughly equal ratios [41]. This could indicate that inorganic arsenic was lost readily during washing. Soaking for 30 min reduced the arsenic content by 45–69% for *L. digitata* and *S. japonica* [41] and soaking for 15 min reduced 28% of arsenic from *H. fusiforme* [39]. According to the literature, the most successful method of arsenic reduction is soaking of dried samples followed by boiling, which removed around 90% of arsenic from *L. digitata* and *S. japonica* [41], but only 28% from *U. pinnatifida* [39].

In all presented studies where inorganic arsenic was analyzed, the amount (mg/kg dw) of inorganic arsenic decreased after processing compared to before processing. It was not consistent whether the inorganic or organic fraction of arsenic was lost more readily during cooking. Thus, it cannot be concluded that inorganic arsenic, which is considered the most toxic, is reduced to a greater extent than organic arsenic.

The reduction of arsenic during processing may depend on the species of seaweed, the type and extent of the applied processing and conditions used, molecular weight and solubility of the arsenic species present, as well as where they are localized in the seaweed tissue. For arsenic reduction, it is also crucial that any water used contains low amounts of arsenic.

As described in the case of iodine, the arsenic content has previously been shown to vary with different thallus parts, but not in a consistent manner. Pétursdóttir and Blagden et al. [57] found a higher content of total arsenic in the blades and holdfasts of *S. latissima* than in the stipes. The opposite finding was reported by Kreissig and Hansen et al. [53], with higher total arsenic content in stipes than in blades of *S. latissima*. Based on this, our removal of the stipe and holdfast prior to processing might have influenced the reduction in arsenic content found during processing in this study, but it is not possible to predict in which direction since the findings from literature point in opposite directions.

#### 4.1.3. Cadmium

**Figure 2:**
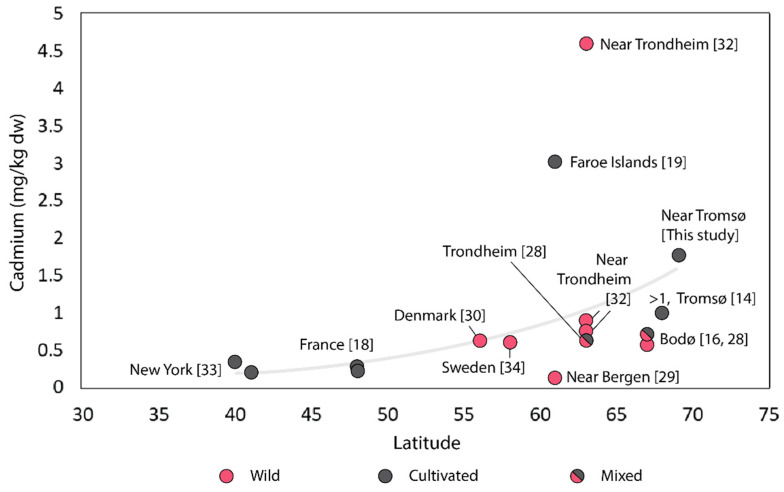
Cadmium content in wild and cultivated *S. latissima* grown at latitudes ranging from 40–69° N. Prepared using original data and data from the indicated literature.

The content of cadmium in *S. latissima* cultivated in this study was 2.00 ± 0.41 mg/kg dw (weighted mean), with results ranging from 1.23–2.64 mg/kg dw. Cadmium concentrations between 0.13–4.6 mg/kg dw have been reported previously for *S. latissima* [12,14,15,27,28,29,31,32,33]. In *S. latissima* from Norway, the content varied from very low (0.13 mg/kg dw) in wild kelp from Solund (between Bergen and Ålesund) to very high levels (4.6 mg/kg dw) in wild kelp from Vanvikan (near Trondheim; Figure 2). In Figure 2, the literature values of cadmium versus the latitude where the samples were grown have been plotted. There is a tendency towards higher cadmium content in samples from higher latitudes. However, there are several outliers from the curve, indicating that factors other than latitude also influence the result. It has been reported previously that samples from the north of Norway (68–70° N) have a higher cadmium content (>1 mg/kg dw) than samples from more southerly parts of Norway [10]. In the present study, we also found cadmium contents above 2 mg/kg dw in *S. latissima* cultivated near Tromsø. To the best of our knowledge, it is not known why this relationship exists and which other factors influence the content of cadmium in *S. latissima*. A more thorough investigation into these phenomena is needed. As for the differences between wild and cultivated sugar kelp, it should be noted that a direct comparison based on the data presented in Figure 2 should be performed with caution, since data were included without discrimination based on harvesting month, age of thallus, or other factors. Whereas cultivated kelp is usually sampled when they are ripe (May–June), wild kelp is often sampled for biomonitoring purposes and thus sampled throughout the year. Since there may be seasonal variations in cadmium content [34], as well as differences between young and old thallus tissue [58,59], these factors should be taken into account for comparisons between wild and cultivated kelp.

Cadmium may be difficult to extract using traditional processing technology, as it can be bound to the complex alginate structure of brown algae. A cation exchange process is responsible for the uptake of cadmium into alginate [60]. Some research has also suggested that cadmium may bind to fucans [61]. The complex nature of the binding may render it difficult to remove cadmium during processing, except if the concept of ion exchange is applied or the algal structure is partly degraded. Stévant and Marfaing et al. [14] showed that cadmium can be released from *Alaria esculenta* when soaked in a 2.0 M sodium chloride (NaCl) solution, but not when soaked in 0.5 and 1.0 M NaCl solutions. Thus, cadmium can be released in a cation exchange mechanism if the conditions are favorable. In another study, the content of cadmium was significantly reduced by a combination of boiling and fermentation (−35%), but not by boiling alone [15]. This may result from partial degradation of the seaweed during the fermentation process. In the present study, the content of cadmium did not change during rinsing and boiling in water—processes that do not degrade the algal structure or provide favorable conditions for ion exchange. More research into how cadmium levels can be reduced in kelp while still providing a satisfactory food product is needed, especially if seaweeds high in cadmium in their raw state are to be consumed.

#### 4.1.4. Lead

The weighted mean concentration of lead was 0.852 ± 0.878 mg/kg dw for the analyzed samples, with values ranging from 0.292–3.37 mg/kg dw. This is in line with previous findings, which vary between 0.18–1.66 mg lead/kg dw for *S. latissima* [12,15,27,28,29,31,32,33], although some data points in the present study were above this range. In a thorough report presenting the results from 148 samples of *S. latissima*, an analytical range of <0.22–5.7 was presented [10]. Lead is said to be the least problematic PTE with regards to seafood, and seaweed has the same content as other fishery products [62]. According to Duinker and Kleppe et al. [10], the level of lead in macroalgae is generally low. Perhaps as a result of this, several studies investigating the effect of processing on the content of PTEs in *S. latissima* and other brown algae exclude analysis of lead and only three other studies were found where the lead content of brown algae after processing was reported.

Like for cadmium, no lead reduction occurred during rinsing or boiling in this study. The same has been documented in a previous study using *S. latissima* from the Faroe Islands, to which both boiling (15 min) and fermentation were applied without significantly altering the lead content of the samples [15]. During a study of two lesser known brown algae, *Cystoseira abies-marina* and *Zonaria tournefortii*, no lead was found in the former species in the raw state nor after processing (<limit of quantification), whereas for the latter species, the lead content remained unchanged during rehydration in milli-Q water but increased significantly during steaming [63]. Contrary to this, the following study has documented loss of lead during processing. Ownsworth and Selby et al. [41] studied the effect of cooking treatments (rehydration, blanching, and rehydration followed by bringing to a boil) on the content of lead in two samples of *Laminaria digitata*, where one of the samples was washed and the other unwashed and one commercial sample of *S. japonica*. They found that the content of lead in the unwashed *L. digitata* sample decreased significantly from around 0.16 to 0.02–0.04 mg/kg dw for all treatments. In contrast, the washed sample had an initial content of around 0.04 mg/kg dw and remained in the range of 0.02–0.04 mg/kg dw after all treatments. Thus, it seems that in this case, washing reduced the content of lead, whereas subsequent treatments did not. The lead content of the *S. japonica* sample also decreased during processing, but to a lesser degree than the unwashed *L. digitata* sample. It is not known whether the *S. japonica* sample was washed prior to commercialization.

#### 4.1.5. Mercury

The concentration of mercury in brown algae is generally quite low, with values between below the detection limit to 0.1 mg/kg dw having been reported for *S. latissima* [10,12,15,27,28,31,32,33]. In the present study, a content of 0.0323 ± 0.0022 mg/kg dw was found in raw kelp, which is well within the previously reported range. The chemical form of mercury determines its toxicity, and methylmercury (MeHg) is considered the most toxic when administered orally. To the best of our knowledge, the content of MeHg in *S. latissima* has only been assessed in one study, where a content below the limit of detection (<0.01 mg/kg dw) was found [28]. Methyl mercury and inorganic mercury (Hg(II)) have been quantified in other brown algal species but in low amounts. *Undaria pinnatifida* and *H. fusiforme* were found to contain 0.01 mg/kg MeHg and 0.06 mg/kg Hg(II), while *Himanthalia elongata* contained 0.11 mg/kg MeHg and 0.06 mg/kg Hg(II) [64].

Since the content of mercury in seaweeds is not considered problematic, the analysis of mercury has typically been excluded during investigations of reduction of PTEs during processing. In this study, the mercury content was significantly increased after rinsing (+31%). The content after boiling was, on the other hand, not significantly different from the content in raw kelp and the final concentration of lead was 13% lower than in raw kelp. This result is comparable to the findings of a previous study, where the content of mercury in *S. latissima* was significantly reduced by a combination of boiling and fermentation (−35%), but not by boiling alone [15].

### 4.2. Tolerable Daily Amounts of Saccharina latissima

The limiting factor for consumption of *S. latissima* is the iodine content. The daily required amount of iodine for non-pregnant, non-lactating adults is 150 μg, and a maximum limit of 600 μg is set in Europe. For pregnant and lactating women, the requirement is slightly elevated, while the recommended maximum intake remains the same.

For dried raw sugar kelp, only 0.04 g is needed to reach 150 μg of iodine, while 0.15 g provides the maximum recommended intake. After boiling, 0.25 g provides the daily requirement and 1 g provides the maximum recommended intake. Since a safe consumption of *S. latissima* is limited by its iodine content, the contents of arsenic, cadmium, lead, and mercury are discussed assuming that the consumption remains at or below 1 g boiled, dry sugar kelp.

A daily consumption of a maximum of 1 g of boiled, dry, sugar kelp will provide a maximum of 3.0% of TDI of inorganic arsenic, 8.8% of TDI of cadmium, 3.3% of TDI of lead, and 0.2% of TDI of mercury for a 70 kg person. A total consumption of PTEs above the TDI thus requires that the diet already contains >90% of TDI of cadmium, >96% of TDI of lead, >97% of TDI of inorganic arsenic, or >99% of TDI of mercury. Therefore, as long as the remaining part of the diet contains arsenic, cadmium, lead, and mercury below these limits, adding a maximum of 1 g of boiled, dried sugar kelp should not pose a risk of toxicity. However, for light weight individuals, including children, extra caution should be exercised.

### 4.3. Commercial Impact

Cultivation and processing strategies for kelp that result in a controlled iodine content and food that is safe to consume is crucial for the commercial utilization of kelp as human food. Limiting the daily recommended consumption to e.g., 0.25 or 1 g, as discussed here, is a major obstacle in most markets. However, for some foods the seaweed is used as salt replacement or a spice, and even small amounts (e.g., 1 g) may therefore be of interest. The major processing step in this respect is the boiling, leading to both energy costs, investment in boiler tanks, and handling costs upon industrial implementation. As shown by Nielsen and Holdt et al. [16], the required iodine reduction may be achieved even by blanching at temperatures as low as 45 °C. Such temperatures may be generated at considerably lower energy consumption than boiling by use of heat pumps and waste heat. The economic feasibility of such processing should be investigated, taking into consideration the effect of boiling/blanching on other aspects of sugar kelp quality such as color, texture, and microbiological safety [65], as well as alterations in nutrient and taste components [16]. For domestic use, the processing conditions presented in this paper can easily be applied, and the instructions can, for instance, be provided on the packaging if the kelp is sold frozen.

## 5. Conclusions

This study provides data on the effect of cultivation depth on the reduction in the content of PTEs in *S. latissima* after processing. Furthermore, to the best of our knowledge, this study reports contents of iodine, arsenic, cadmium, lead, and mercury in a more northern location in Norway than previous studies investigating cultivated sugar kelp.

Eighty five percent of iodine was removed on average during rinsing and boiling of *S. latissima* for 15 min. Kelp cultivated at a shallower depth (1 m) contained less iodine compared to deeper cultivation depths (6 and 9 m), but the effect disappeared after processing. Since the final content of iodine in the kelp was not dependent on the cultivation depth, the type and extent of processing determines if cultivation depth should be considered as a factor in cultivation infrastructure design and implementation, or alternatively, in product segmentation.

Processing decreased the arsenic content but not the content of cadmium, mercury, or lead. Rinsed and boiled kelp showed a significantly lower arsenic content than raw sugar kelp. Cultivation depth did not have a statistically significant effect on the content of arsenic, cadmium, lead, or mercury.

To reach the daily required intake of iodine (150 μg), 0.25 g of boiled, dried sugar kelp can be consumed. The maximum recommended intake (600 μg) amounts to 1 g of boiled, dried kelp. This amount provides <9% of the TDI of arsenic, cadmium, lead, and mercury for a 70 kg person. If consumed in this range, toxic effects are not likely unless the diet already contains >90% of the TDI of the PTEs.

## Figures and Tables

**Table 1:** The content of PTEs (mg/kg dry weight) and the dry matter content (%) of dried samples of *Saccharina latissima* cultivated at 0–2, 5–7, and 8–10 m in raw, rinsed, and boiled state. The number of analytical replicates were: Iodine (*n* = 3); dry matter (*n* = 2); arsenic, cadmium, lead, and mercury (*n* = 1). In addition to analytical replicates, sample preparation was conducted in duplicate for processed samples, and thus the number of total parallels was doubled for processed samples (*n* = 6, 4, or 2) compared to raw samples (*n* = 3 or 1). The data are presented as mean ± standard deviation.

Element	Processing Step	All Depths	0–2 m	5–7 m	8–10 m
Iodine (I)	Raw	4100 ± 700 ^A^	3300 ± 0 ^Aa^	3600 ± 100 ^Ab^	4700 ± 100 ^Ac^
	Rinsed	3600 ± 400 ^A^	3200 ± 100 ^Aa^	3400 ± 200 ^Ba^	4100 ± 200 ^Bb^
	Boiled	600 ± 100 ^B^	600 ± 100 ^Ba^	600 ± 100 ^Ca^	600 ± 100 ^Ca^
Arsenic (As)	Raw	62.7 ± 4.3 ^A^	57.7	64.9	65.4
	Rinsed	50.8 ± 3.8 ^B^	49.8 ± 6.4 ^a^	50.2 ± 2.5 ^a^	52.5 ± 3.9 ^a^
	Boiled	36.0 ± 3.1 ^B^	34.2 ± 0.8 ^a^	39.5 ± 1.1 ^a^	34.4 ± 3.1 ^a^
Cadmium (Cd)	Raw	1.77 ± 0.50 ^A^	1.23	1.89	2.20
	Rinsed	2.05 ± 0.39 ^A^	1.83 ± 0.16 ^a^	1.83 ± 0.37 ^a^	2.50 ± 0.04 ^a^
	Boiled	2.19 ± 0.38 ^A^	1.76 ± 0.00 ^a^	2.28 ± 0.33 ^a^	2.52 ± 0.17 ^a^
Mercury (Hg)	Raw	0.0323 ± 0.0022 ^A^	0.0298	0.0328	0.0342
	Rinsed	0.0424 ± 0.0037 ^B^	0.0441 ± 0.0072 ^a^	0.0416 ± 0.0016 ^a^	0.0414 ± 0.0023 ^a^
	Boiled	0.0280 ± 0.0032 ^A^	0.0278 ± 0.0006 ^a^	0.0284 ± 0.0052 ^a^	0.0277 ± 0.0047 ^a^
Lead (Pb)	Raw	0.446 ± 0.178 ^A^	0.695	0.351	0.292
	Rinsed	0.946 ± 0.731 ^A^	0.695 ± 0.202 ^a^	1.48 ± 1.46 ^a^	0.665 ± 0.439 ^a^
	Boiled	1.16 ± 1.14 ^A^	0.727 ± 0.135 ^a^	1.93 ± 2.02 ^a^	0.83 ± 0.72 ^a^
Dry matter	Rinsed	89.1 ± 0.3	89.2 ± 0.2	89.1 ± 0.2	88.8 ± 0.3
	Boiled	87.2 ± 0.3	87.0 ± 0.1	87.3 ± 0.4	87.2 ± 0.3

Different capital letters indicate significant differences within the columns (between processes) for each element (I, As, Cd, Hg, Pb). Different lower-case letters indicate significant differences within the rows (between depths).

**Table 2:** Amount (in g dry weight) of *S. latissima* providing 150 and 600 μg iodine.

Processing Step	150 μg Iodine	600 μg Iodine
Raw	0.037	0.15
Rinsed	0.042	0.17
Boiled	0.25	1.0

**Table 3:** The content of arsenic, cadmium, lead, and mercury in 0.25 and 1 g of boiled, dried *S. latissima* and the tolerable amounts of the PTEs. The tolerable daily and weekly intakes (TDI, TWI) for the PTEs are provided by EFSA [35,36,37,38].

Element	Chemical Form	Content		Tolerable Amounts				TDI (%): 1 g kelp/70 kg bw
				μg/kg bw		μg/70 kg		
		μg/g	μg/0.25 g	TWI	TDI	TWI	TDI	
Cd	Total	2.19	0.55	2.5	0.4	175	25	8.7
Hg	Total	0.03	0.01	-	-	-	-	-
	MeHg	n.a.	n.a.	1.3	0.2	91	13	0.2
	iHg	n.a.	n.a.	4	0.6	280	40	0.1
Pb	Total	1.16	0.29	3.5	0.5	245	35	3.3
As	Total	36.0	9.00	-	-	-	-	-
	iAs ^1^	0.63	0.16	2.1	0.3	147	21	3.0

^1^ iAs was not measured but is based on calculations assuming 1% of total arsenic is inorganic and that no inorganic arsenic was lost during processing. n.a.: not analyzed; -: there are no TDI or TWI for this matrix.

**Table 4:** Reduction in iodine content during processing of *S. latissima* at various water-to-seaweed ratios. Tap water was used in all studies.

Origin	Initial State	Processing	Reduction (%)	Final Content (mg/kg dw)	Water-to-Kelp Ratio	Reference
Faroe Island	Frozen, cut, refrozen	Boiling (15 min)	38.4	1620	3.8	[15]
Norway	Frozen-thawed	Boiling (15 min)	85.6	600	10	This study
France	Fresh, <2 h post-harvest	Soaking (32 °C) w/air bubbling (1–22 h)	84.8	1000	20	[14]
Norway	Fresh, whole thallus	Blanching (30 °C, 300 s)	78.0	1014	33	[16]
		Blanching (45 °C, 300 s)	91.6	388	33	
		Blanching (60 °C, 300 s)	93.0	321	33	
		Blanching (80 °C, 120 s)	93.6	293	33	

**Table 5:** Reduction in total (tAs) and inorganic (iAs) arsenic during processing of brown algae.

Processing	Species	Location	Raw State	Reduction (%)		Final Concentration (mg/kg dw)		Reference
				tAs	iAs	tAs	iAs	
Blanching (10–20 s)	*L. digitata*	Scotland	Washed and dried	29	5	32	19	[41]
	*L. digitata*	Scotland	Dried	39	33	28	12	[41]
	*S. japonica*	Japan	Dry, commercial samples	22	61	58	0.059	[41]
Boiling (15 min)	*S. latissima*	Norway	Frozen-thawed	43	n.a.	36	n.a.	This study
		Faroe Islands		−8.2	n.a.	42	n.a.	[15]
Boiling (20 min)	*H. fusiforme*	Unknown	Dry, commercial samples	46	n.a.	56	n.a.	[39]
	*H. fusiforme*			30	46	91	43	[40]
	*H. fusiforme*			43	50	75	43	[40]
	*H. fusiforme*			33	49	84	45	[40]
	*U. pinnatifida*			−0.34	n.a.	50	n.a.	[39]
	*U. pinnatifida*			49	n.a.	26	n.a.	[56]
Boiling (60 min)	*S. japonica*	Unknown	Dry, commercial samples	65	n.a.	27	n.a.	[56]
Soaking (15 min)	*H. fusiforme*	Unknown	Dry, commercial samples	28	n.a.	94	n.a.	[39]
Soaking (30 min)	*L. digitata*	Scotland	Dried	69	64	14	6.4	[41]
	*S. japonica*	Japan	Dry, commercial samples	45	67	41	0.050	[41]
Soaking (15 min), followed by boiling (2 min)	*U. pinnatifida*	Unknown	Dry, commercial samples	28	n.a.	34	n.a.	[39]
Soaking (30 min), followed by bringing to boil	*L. digitata*	Scotland	Washed and dried	87	95	5.9	1.0	[41]
	*L. digitata*	Scotland	Dried	91	96	4.0	0.72	[41]
	*S. japonica*	Japan	Dry, commercial samples	87	75	9.4	0.038	[41]
Boiling (15 min), lactic acid fermentation (48 h)	*S. latissima*	Faroe Islands	Frozen-thawed	5.9	n.a.	37	n.a.	[15]

n.a.: not analyzed.

## Data Availability

The data presented in this study are available on request from the corresponding author.
